# SGLT2 inhibitors in T2D and associated comorbidities — differentiating within the class

**DOI:** 10.1186/s12902-019-0387-y

**Published:** 2019-06-17

**Authors:** Guntram Schernthaner, Heinz Drexel, Evgeny Moshkovich, Birute Zilaitiene, Emil Martinka, Leszek Czupryniak, Tamás Várkonyi, Andrej Janež, Kristine Ducena, Katarina Lalić, Tsvetalina Tankova, Martin Prázný, Lea Smirčić Duvnjak, Olga Sukhareva, Harald Sourij

**Affiliations:** 1 Department of Medicine I, Rudolfstitung Hospital, Vienna, Austria; 2 VIVIT-Institute, Academic Teaching Hospital Feldkirch, Feldkirch, Austria; 3 Unit of Endocrinology and Metabolism, Sapir Medical Center, Kfar-Saba, Israel; 4 Institute of Endocrinology, Lithuanian University of Health Sciences, Kaunas, Lithuania; 5 National Institute of Endocrinology and Diabetology, Lubochna, Slovakia; 6 Department of Diabetology and Internal Medicine, Warsaw Medical University, Warsaw, Poland; 7 1st Dept of Internal Medicine, University of Szeged, Szeged, Hungary; 8 Department of Endocrinology, Diabetes and Metabolic Diseases, University Medical Centre, Ljubljana, Slovenia; 9 Division of Endocrinology, Faculty of Internal Medicine, University of Latvia, Riga, Latvia; 10 Clinic for Endocrinology, Diabetes and Metabolic Diseases, Clinical Centre of Serbia, Faculty of Medicine, University of Belgrade, Beograd, Serbia; 11 Clinical Centre of Endocrinology, Medical University – Sofia, Sofia, Bulgaria; 12 Diabetes Centre, Charles University and General Faculty Hospital, Prague, Czech Republic; 13 School of Medicine, University of Zagreb, Vuk Vrhovac University Clinic-UH Merkur, Zagreb, Croatia; 14 Endocrinology Research Centre, Moscow, Russian Federation; 15 Division of Endocrinology and Diabetology, Medical University of Graz, Graz, Austria; 16 Vorarlberg Institute for Vascular Investigation and Treatment (VIVIT), Feldkirch, Austria; 17 Division of Angiology, Swiss Cardiovascular Center, University Hospital of Berne, Bern, Switzerland; 18 Private University of the Principality of Liechtenstein, Triesen, Liechtenstein; 19 Drexel University College of Medicine, Philadelphia, PA USA

**Keywords:** Type 2 diabetes, SGLT2 inhibitor, Empagliflozin, Canagliflozin, Dapagliflozin, Cardiovascular disease

## Abstract

**Background:**

For patients with type 2 diabetes (T2D), cardiovascular disease (CVD) is the single most common cause of mortality. In 2008 and 2012, the Federal Drug Administration (FDA) and the European Medicines Agency (EMA) respectively mandated cardiovascular outcomes trials (CVOTs) on all new anti-diabetic agents, as prospective trials statistically powered to rule out excess cardiovascular risk in patients with T2D. Unexpectedly, some of these CVOTs have demonstrated not only cardiovascular safety, but also cardioprotective effects, as was first shown for the SGLT2 inhibitor empagliflozin in EMPA-REG OUTCOME.

**Expert opinion:**

To debate newly available CVOT data and to put them into context, we convened as a group of medical experts from the Central and Eastern European Region. Here we describe our discussions, focusing on the conclusions we can draw from EMPA-REG OUTCOME and other SGLT2 inhibitor CVOTs, including when considered alongside real-world evidence.

**Conclusion:**

CVOTs investigating SGLT2 inhibitors have suggested benefits beyond glucose lowering that have been confirmed in real-world evidence studies.

## Background

The majority of people worldwide who are living with diabetes are affected by type 2 diabetes (T2D) [1, 2] and, among these individuals, more than 50% of mortality is due to cardiovascular (CV) causes [3]. Estimates of 6 or 12 fewer years of life for a typical 60-year old male with T2D or with T2D and CV disease (CVD) have been given when compared with counterparts in the non-diabetic population [4]. Furthermore, the presence of T2D confers a 2- to 5-fold higher risk of developing heart failure (HF) and a 60–80% greater probability of death from CV causes in those who have established HF [5–8]. As with CVD, kidney disease is a strong predictor of mortality in people with T2D [9], and up to 40% of people with T2D will eventually develop kidney failure [10]. Both low estimated glomerular filtration rate (eGFR) and high urine albumin-to-creatinine ratio (UACR) are independent predictors of CV death [11]. Thus, the morbidity and mortality burdens presented by CV and renal complications of T2D are considerable.

Although lifestyle interventions are a key first step in managing the care of people with T2D, the majority of patients will eventually need medication [12]. Before the late 1950s, when biguanides were introduced, only insulin and sulfonylureas were available, but from the 1980s onwards metformin quickly became the glucose-lowering drug of choice for people with T2D [13]. Indeed, unless a specific contraindication such as severe renal or liver disease is present, metformin remains the first-line drug for the treatment of people with T2D [13]. Although three new classes of T2D agents were introduced in the 1990s (α-glucosidase inhibitors, meglitinides and thiazolidinediones), it was not until the turn of the Twenty-First Century that the so-called “newer T2D agents” were introduced: dipeptidyl peptidase-4 (DPP-4) inhibitors, glucagon-like peptide-1 (GLP-1) receptor agonists and sodium–glucose transporter 2 (SGLT2) inhibitors [13].

In recent years, the Federal Drug Administration (FDA) has mandated CV outcomes trials (CVOTs) to assess the CV safety of all new glucose-lowering drugs, while the European Medicines Agency (EMA) has recommended either a CVOT or a meta-analysis [14, 15]. Unexpectedly, the results reported for treatment with the SGLT2 inhibitor empagliflozin in the EMPA-REG OUTCOME CVOT showed, for the first time, that an anti-diabetic agent could not only deliver glucose-lowering efficacy without any additional CV risk, but could actually provide CV benefit [16]. This benefit included a reduction in CV death in the study population, which also contributed to a reduced risk of death by any cause [16]. Subsequent CVOTs have also revealed CV benefits for a small number of other glucose-lowering drugs, whereas others did not show any CV benefits [16–26]. Among CV benefits, only SGLT2 inhibitors have suggested a decrease in hospitalisation for heart failure [6, 16–18].

With the new CVOT results, a paradigm for anti-diabetic drugs is emerging in which glucose-lowering is only one element of the overall treatment aim. As CV risk is the aspect of T2D that leads to the greatest mortality [3], we believe that CV health is an important consideration when deciding on the most appropriate therapies for any one individual. An integrated approach to disease management is desirable, encompassing prevention or control of CV risk together with the avoidance of renal complications, as these two factors are inextricably linked [27]. Furthermore, drug dosing can be challenging for patients who develop chronic kidney disease (CKD), as impaired kidney function can potentially influence the pharmacokinetics of every therapeutic agent, and through different mechanisms [28]. Given that many glucose-lowering drugs have not yet been extensively tested in a CKD population, making detailed analyses of renal data in the CVOT studies could be especially important, although dedicated renal studies are also ongoing.

At the American Diabetes Association (ADA) annual congress in June 2017, several new sets of data were presented from studies of SGLT2 inhibitors, including the main results from the CANVAS Program on canagliflozin and the CVD-REAL real-world evidence (RWE) study encompassing empagliflozin, canagliflozin and dapagliflozin [17, 29]. As a group of experts in the field from the Central and Eastern European Region, we subsequently met to discuss the significance of the results and to put them into context for practitioners treating people with T2D. We here report the resultant discussions on how best to interpret EMPA-REG OUTCOME, the CANVAS Program and RWE in order to inform clinical practice. During the preparation of this manuscript, important disclosures were made regarding the results of DECLARE-TIMI 58 (a CVOT on dapagliflozin) and regarding early results of the EMPRISE real-world evidence study on empagliflozin [18, 30]; owing to their importance for understanding the broader picture of SGLT2 inhibitor CVOTs, these new disclosures are also briefly discussed.

## Expert opinion

### SGLT2 inhibitors

SGLT2 inhibitors partially block the reabsorption of glucose in the proximal tubules in the kidney [31]. The members of this class have somewhat similar molecular structure but nevertheless possess differing relative selectivity for SGLT2 compared with SGLT1 [32]. Until recently, three SGLT2 inhibitors had been approved by the EMA and FDA for the treatment of T2D, as an adjunct to diet and exercise: canagliflozin (approval: EMA/FDA 2013); dapagliflozin (approval: EMA 2012/FDA 2014); and empagliflozin (approval: EMA/FDA 2014). Of these three, empagliflozin shows the highest relative selectivity, being more than 2500-fold more selective for SGLT2 than SGLT1, followed by dapagliflozin at > 1100-fold, and canagliflozin at > 250-fold [32]. Such factors may be pertinent when discussing relative efficacies and safety profiles of these molecules, although the clinical relevance is unknown.

At the present time, CVOTs have been reported for empagliflozin (EMPA-REG OUTCOME), canagliflozin (the CANVAS Program) and dapagliflozin (DECLARE-TIMI 58) (Table 1). An additional SGLT2 inhibitor, ertugliflozin, was approved by the EMA and FDA in 2018 and 2017, respectively; however, results have not yet been reported for the ongoing ertugliflozin CVOT (VERTIS-CV) (Table 1).

**Table 1:** Overview of SGLT2 inhibitor CVOTs

Study	Trial #	Completion	Primary outcome(s)	Main reported secondary CV and renal outcomes
EMPA-REG OUTCOME (empagliflozin) [16, 33]	NCT01131676	Completed 2015; results published	Time to first occurrence of 3P-MACE	4P-MACE; CV death; death by any cause; symptomatic MI; symptomatic non-fatal MI; silent MI; hospitalisation for unstable angina; coronary revascularisation procedure; stroke; non-fatal stroke; TIA; HHF; HHF or CV death excluding stroke; incident or worsening nephropathy or CV death; incident or worsening nephropathy; progression to macroalbuminuria; dSCr and eGFR ≤45 ml/min/1.73m^2^; initiation of RRT; dSCr and eGFR ≤45 ml/min/1.73m^2^, initiation of RRT or renal death; incident albuminuria in patients with normal albumin at baseline
CANVAS Program (canagliflozin) [17, 34]	NCT01032629 (CANVAS) & NCT01989754 (CANVAS-R)	Completed 2017; results published	Time to first occurrence of 3P-MACE	Death by any cause; CV death; progression of albuminuria; CV death or HHF; non-fatal MI; non-fatal stroke; MI; stroke; hospitalisation for any cause; HHF; new-onset albuminuria; new-onset microalbuminuria; new-onset macroalbuminuria; dSCr, ESRD or renal death; dSCr, ESRD, renal death or new-onset macroalbuminuria; dSCr, ESRD, renal death or CV death; ≥40% decrease in eGFR, ESRD or renal death; ≥40% decrease in eGFR, ESRD, renal death or new-onset macroalbuminuria; ≥40% decrease in eGFR, ESRD, renal death or CV death; 40% reduction in eGFR; dSCr; ESRD; ESRD or renal death
DECLARE-TIMI 58 (dapagliflozin) [18]	NCT01730534	Completed 2018; results published	Time to first occurrence of 3P-MACE Time to first occurrence of CV death or HHF	≥40% decrease in eGFR to eGFR < 60 ml/min/1.73m^2^, ESRD, renal death or CV death; death by any cause; ≥40% decrease in eGFR to eGFR < 60 ml/min/1.73m^2^, ESRD or renal death; HHF; MI; ischaemic stroke; CV death; non-CV death
VERTIS-CV (ertugliflozin) [35]	NCT01986881	Estimated completion 2019	Time to first occurrence of 3P-MACE	Results not yet reported

Definitions differed between trials. 3P-MACE is a composite of CV death, MI and stroke. 4P-MACE is a composite of CV death, MI, stroke and hospitalisation for unstable angina. Study names: EMPA-REG OUTCOME [cardiovascular outcomes trial of empagliflozin]; CANVAS, Canagliflozin Cardiovascular Assessment Study; CANVAS-R, Study of the Effects of Canagliflozin on Renal Endpoints in Adult Subjects with T2DM; DECLARE-TIMI, Multicenter Trial to Evaluate the Effect of Dapagliflozin on the Incidence of Cardiovascular Events; VERTIS-CV Randomized, Double-Blind, Placebo-Controlled, Parallel-Group Study to Assess Cardiovascular Outcomes Following Treatment With Ertugliflozin (MK-8835/PF-04971729) in Subjects With Type 2 Diabetes Mellitus and Established Vascular Disease. 3/4P-MACE, 3/4-point major adverse CV event; CV, cardiovascular; dSCr, doubling of serum creatinine; eGFR, estimated glomerular filtration rate; ESRD, end-stage renal disease; HHF, hospitalisation for heart failure; MI, myocardial infarction; RRT, renal replacement therapy; TIA, transient ischaemic attack

### EMPA-REG OUTCOME

The EMPA-REG OUTCOME CVOT investigated empagliflozin in addition to standard of care in a population of 7200 adult patients with both T2D and established CVD at baseline, defined as one or more of previous myocardial infarction (MI), stroke or unstable angina; multivessel coronary artery disease (CAD); single-vessel CAD if in addition to positive stress ischaemia test or recent hospitalisation for unstable angina; or occlusive peripheral artery disease (PAD) [16]. As the study drug or placebo was added to standard of care, patients in all study arms were well treated for dyslipidaemia and hypertension [16].

The primary composite outcome was time to first occurrence of 3-point major adverse CV event (3P-MACE; that is, CV death, non-fatal stroke or non-fatal myocardial infarction). Although EMPA-REG OUTCOME was only designed to test non-inferiority for this outcome, the study unexpectedly also demonstrated superiority, with a relative risk reduction (RRR) of 14%, primarily driven by a 38% reduction in CV death [16]. Thus, key outcomes in EMPA-REG OUTCOME were significantly improved when empagliflozin was added to standard of care (Table 2).

**Table 2:** Key efficacy outcomes in SGLT2 inhibitor CVOTs

	EMPA-REG OUTCOME [16, 33]		CANVAS Program [17, 34]		DECLARE-TIMI 58 [18]	
	Placebo (*N* = 2333)	Empagliflozin (*N* = 4687)	Placebo (*N* = 4347)	Canagliflozin (*N* = 5795)	Placebo (*N* = 8578)	Dapagliflozin (*N* = 8582)
3P-MACE (CV death, MI or stroke)						
Rate per 1000 pt-yrs	43.9	37.4	31.5	26.9	24.2	22.6
HR (95% CI; *p*-value)	0.86 (0.74–0.99; *p* = 0.04)		0.86 (0.75–0.97; *p* = 0.02)		0.93 (0.84–1.03; *p* = 0.17)	
RRR (ARR)	14% (6.5 events/1000 pt-ys)		14% (4.6 events/1000 pt-yrs)		N/A	
CV death						
Rate per 1000 pt-yrs	20.2	12.4	12.8	11.6	7.1	7.0
HR (95% CI; *p*-value)	0.62 (0.49–0.77; *p* < 0.001)		0.87 (0.72–1.06)†		0.98 (0.82–1.17)†	
RRR (ARR)	38% (7.8 events/1000 pt-yrs)		N/A		N/A	
Death by any cause						
Rate per 1000 pt-yrs	28.6	19.4	19.5	17.3	16.4	15.1
HR (95% CI; *p*-value)	0.68 (0.57–0.82; *p* < 0.001)		0.87 (0.74–1.01; *p* = 0.24)		0.93 (0.82–1.04)†	
RRR (ARR)	32% (9.2 events/1000 pt-yrs)		N/A		N/A	
HHF						
Rate per 1000 pt-yrs	14.5	9.4	8.7	5.5	8.5	6.2
HR (95% CI; *p*-value)	0.65 (0.50–0.85; *p* = 0.002)		0.67 (0.52–0.87)†		0.73 (0.61–0.88)†	
RRR (ARR)	35% (5.1 events/1000 pt-yrs)		33% (3.2 events/1000 pt-yrs)		27% (2.3 events/1000 pt-yrs)	
Renal composite (renal function decline*, ESRD or renal death)						
Rate per 1000 pt-yrs	11.5	6.3	9.0	5.5	7.0	3.7
HR (95% CI; *p*-value)	0.54 (0.40–0.75; *p* < 0.001)†		0.60 (0.47–0.77)†		0.53 (0.43–0.66)†	
RRR (ARR)	46% (5.2 events/1000 pt-yrs)		40% (3.5 events/1000 pt-yrs)		47% (3.3 events/1000 pt-yrs)	
Alternative renal composite (progression to macroalbuminuria, dSCr, ESRD or renal death)						
Rate per 1000 pt-yrs	76.0	47.8	27.4	15.1	Not reported	
HR (95% CI; *p*-value)	0.61 (0.53–0.70; *p* < 0.001)		0.58 (0.50–0.67)†			
RRR (ARR)	39% (28.2 events/1000 pt-yrs)		42% (12.3 events/1000 pt-yrs)			

Please note that direct comparison of trials may not be accurate owing to differences in study design, populations and methodology. RRR and ARR are only shown where a significant reduction was reported, or a nominally significant reduction in the case of an exploratory analysis. *Defined as: dSCr accompanied by eGFR of < 45 ml/min/1.73 m^2^ in EMPA-REG OUTCOME; ≤40% decrease in eGFR in the CANVAS Program; and ≤ 40% decrease in eGFR to < 60 ml/min/1.73 m^2^ in DECLARE-TIMI 58. †Exploratory analysis, *p*-value is nominal or not available. 3P-MACE, 3-point major adverse CV event; ARR, absolute risk reduction; CI, confidence interval; CV, cardiovascular; dSCr, doubling of serum creatinine; HHF, hospitalisation for heart failure; HR, hazard ratio; MI, myocardial infarction; pt-yrs, patient-years; RRR, relative risk reduction

Several secondary CV outcomes also showed reductions, including hospitalisation for HF (HHF) by 35% [16]. Death by any cause showed a 32% reduction, and the number needed to treat (NNT) to prevent one death over three years of the study was calculated as 39 [16]. However, no significant differences to placebo were seen for non-fatal stroke or non-fatal MI [16].

#### Empagliflozin in subjects at increased CV risk

It is important to consider which individuals with T2D will benefit from empagliflozin, although, in our opinion, it might be more appropriate simply to exclude those who would not benefit, as empagliflozin is currently somewhat unique in its safety and efficacy profile. We believe that empagliflozin is increasingly perceived as fulfilling a dual role of treating both hyperglycaemia and CV risk factors and is therefore suitable for patients with both T2D and CVD; indeed, this has now been recognised in updated product labels and international guidelines [36–41]. Although the EMPA-REG OUTCOME study included patients with established CVD, a meta-analysis of eight randomised controlled trials (RCTs) of empagliflozin analysed data from 11, 292 subjects, including those at low and medium, as well as high, risk of CV events [42]. The primary endpoint was a composite of CV death, non-fatal MI, non-fatal stroke and hospitalisation for unstable angina (4P-MACE), and there was a secondary endpoint of 3P-MACE [42]. 4P-MACE occurred in 365 (9.5%) patients receiving placebo and 635 (8.5%) patients receiving empagliflozin (HR 0.86; 95% CI 0.76–0.98). 3P-MACE occurred in 307 (8.0%) patients receiving placebo and 522 (7.0%) patients receiving empagliflozin (HR 0.84; 95% CI 0.73–0.96) [42]. It can be inferred from these data that empagliflozin remains associated with a reduced risk of CV morbidity and mortality in patients with T2D, even when those at low/medium CV risk are included in the analysed population.

An analysis of HHF outcomes (a prespecified secondary endpoint) in EMPA-REG OUTCOME showed that the benefits of empagliflozin were consistent in subjects both with and without baseline HF, i.e. primary prevention in those with no HF at baseline and secondary prevention in those for whom HF had been reported at baseline. The analysis showed that 1.8% of patients receiving empagliflozin without HF at baseline experienced an event compared with 3.1% for placebo (HR 0.6; 95% CI 0.43–0.82); for patients with baseline HF, the HHF figures were 10.4 and 12.3%, respectively (HR 0.75; 95% CI 0.48–1.19) [16, 43]. The evidence for primary prevention of HF with empagliflozin has now been recognised by new American Heart Association (AHA) and American College of Cardiology (ACC) guidelines [40], and ongoing clinical studies are seeking to shed more light on this potential benefit; however, it should be noted that empagliflozin is not currently indicated for the treatment of HF.

Reductions in CV death were similar in patients with and without HF at baseline: CV death events occurred in 3.2% of subjects treated with empagliflozin vs 5.3% with placebo (HR 0.60; 95% CI 0.47–0.77) for no baseline HF, and 8.2% vs 11.1% (HR 0.71; 95% CI 0.43–1.16) for subjects with baseline HF [43]. We note that EMPRISE will examine CV death outcomes in a broad CV risk population in routine clinical practice, but these data have not yet been reported [30].

#### Renal outcomes in EMPA-REG OUTCOME

The population of EMPA-REG OUTCOME included a substantial renal burden, with eGFR < 60 mL/min/1.73m^2^ in 26% of patients and 60–< 90 mL/min/1.73m^2^ in 52% of patients [16, 33]. The main pre-specified renal composite outcome of new or worsening nephropathy (progression to macroalbuminuria, doubling of the serum creatinine level, initiation of renal-replacement therapy, or death from renal disease) was significantly reduced by 39%, and doubling of serum creatinine with an eGFR ≤45 mL/min/1.73m^2^ was reduced by 44% when adding empagliflozin to standard of care [16, 33].

Significant benefits were also seen in several other prespecified renal parameters, including a 32% RRR in new or worsening nephropathy or CV death (*p* < 0.001); 38% RRR in progression to macroalbuminuria (*p* < 0.001); and 55% RRR in initiation of renal replacement therapy (*p* = 0.04) [33]. The only exception was incident albuminuria in patients with a normal albumin level at baseline, where no significant difference between study arms was observed [33].

The renal results in EMPA-REG OUTCOME indicate that empagliflozin delays the progression of renal disease when compared with placebo [33]. The change in eGFR over time also supports a nephroprotective effect: after an initial fall when empagliflozin therapy was started, the eGFR of subjects on empagliflozin recovered somewhat and subsequently remained stable, whereas those on placebo demonstrated a steady decline over the period of the study [33]. Furthermore, a post hoc analysis showed that the renal benefits seen with empagliflozin in the full study cohort were consistent in a subgroup of patients with prevalent kidney disease at baseline, defined as having an eGFR < 60 mL/min/1.73m^2^ and/or macroalbuminuria (UACR > 300 mg/g) [44].

Despite the promising renal results, it should be noted that EMPA-REG OUTCOME was a CV trial with 3P-MACE as the primary endpoint, and therefore we await the results of an ongoing dedicated renal study before making a more conclusive assessment of empagliflozin in this setting. Furthermore, in accordance with local prescribing information and licences for SGLT2 inhibitors, if the eGFR is below 60 mL/min/1.73 m^2^ then empagliflozin should not be initiated and must be discontinued if the eGFR persistently falls below 45 ml/min/1.73 m^2^ [36].

#### Key safety data in EMPA-REG OUTCOME

Of note, the only adverse event for which an increased incidence was associated with empagliflozin in EMPA-REG OUTCOME was the occurrence of genital infections (overall, 6.4% vs 1.8%; *p* < 0.001), and these are easily treatable [16]. In contrast to the recent data from the CANVAS Program (see below; Table 3), [17] there was no significant increase with empagliflozin on the risk of bone fracture or lower limb amputation [16, 45], including in patients with PAD [47].

**Table 3:** Key safety outcomes in SGLT2 inhibitor CVOTs

	EMPA-REG OUTCOME [45, 46]*			CANVAS [17, 46]			CANVAS-R [17, 46]		DECLARE-TIMI 58† [18, 46]	
	Placebo (*N* = 2333)	Empa 10 (*N* = 2345)	Empa 25 (*N* = 2342)	Placebo (*N* = 1441)	Cana 100 (*N* = 1445)	Cana 300 (*N* = 1441)	Placebo (*N* = 2903)	Cana 100 (*N* = 2904)	Placebo (*N* = 8569)	Dapagliflozin (*N* = 8574)
Patients with lower limb amputation										
n (%)	43 (1.8)	42 (1.8)	46 (2.0)	22 (1.5)	50 (3.5)	45 (3.1)	25 (0.9)	45 (1.5)	113 (1.3)	123 (1.4)
Events per 1000 patient-years	6.5	6.2	6.8	2.8	6.2	5.5	4.2	7.5	3.3	3.6
Hazard ratio (95% CI)	–	0.96 (0.63–1.47)	1.04 (0.69–1.58)	–	2.24 (1.36–3.69)	2.01 (1.20–3.34)	–	1.80 (1.10–2.93)	–	1.09 (0.84–1.40)
Diabetic ketoacidosis										
	Placebo (*N* = 2333)	Empagliflozin (pooled) (*N* = 4687)		Placebo (pooled‡) (*N* = 4347)			Canagliflozin (pooled‡) (*N* = 5795)		Placebo (*N* = 8569)	Dapagliflozin (*N* = 8574)
n (%)	1 (< 0.1%)	4 (0.1%)		6			12		12	27
Events per 1000 patient-years	< 0.1	0.1		0.3			0.6		0.4	0.9
Hazard ratio (95% CI)	–	1.99 (0.22–17.80)		–			2.33 (1.10–7.17)		–	2.18 (1.10–4.30)

These trials cannot be directly compared, owing to differences in study design, populations and methodology. *Post hoc analysis for lower limb amputation, which was not a prespecified outcome in EMPA-REG OUTCOME. †All amputation; data for lower limb amputation not provided. ‡Diabetic ketoacidosis events pooled across CANVAS and CANVAS-R, and 100 mg and 300 mg doses of canagliflozin. Pooled cohort size indicates the intention to treat population. Cana 100/300, canagliflozin 100/300 mg; Empa 10/25, empagliflozin 10/25 mg

### The CANVAS Program

The CANVAS Program is a pooled analysis of two subsidiary studies: CANVAS, a CV safety study, and CANVAS-Renal (CANVAS-R), which included albuminuria progression as a key outcome alongside CV safety outcomes [17].

#### The CANVAS Program study design

The CANVAS CVOT was a randomised controlled trial designed to assess the CV safety of canagliflozin versus placebo, on top of standard of care in 4330 patients with T2D and either symptomatic CVD (one or more of CAD, cerebrovascular disease or PAD) or multiple CV risk factors (age ≥ 50 and two or more of dyslipidaemia, hypertension, current smoker, ≥10 years diabetes duration, or albuminuria) at baseline [17]. A public disclosure of interim analyses was required for regulatory filings, and plans to further assess CV protection through an expansion of the study were terminated at that time [17, 48, 49]. Instead, in order to achieve sufficient power for CV assessment, the additional CANVAS-R CVOT (*N* = 5813) was combined with CANVAS into the CANVAS Program, enabling a pooled analysis, but excluding events accrued prior to 20 November 2012, which was the date of last unblinding [17, 48, 49]. CANVAS-R was a shorter study, with median follow-up 2.1 years [17].

Statistical analyses were performed as a sequential hypothesis testing plan of the pooled data from CANVAS and CANVAS-R to give a total of 5795 individuals treated with canagliflozin and 4347 placebo controls [17]. As with EMPA-REG OUTCOME, the primary endpoint in the CANVAS Program was time to first occurrence of 3P-MACE (first testing for non-inferiority and then superiority) [17]. Next was a test for superiority for death by any cause, followed by superiority for CV death [17]. Once a non-significant result was encountered, subsequent analyses were exploratory only.

#### Key outcomes in the CANVAS Program

The primary analysis yielded a positive result, with 26.9 participants receiving canagliflozin experiencing an event per 1000 patient-years compared with 31.5 in the placebo group (HR 0.86; 95% CI 0.75–0.97, *p* = 0.02 for superiority); however, superiority was not shown for the first secondary outcome (death by any cause, HR 0.87 95% CI 0.74–1.01), so the sequential hypothesis testing ended there [17]. This means that the results of all subsequent secondary analyses could not be considered as significant but rather as exploratory analyses (Table 2) [17].

It should be noted here that superiority for death by any cause and CV death were additions made by the independent trial steering committee for the CANVAS Program when revising the original analysis plan [48, 49]. These revisions were made in the expectation that the results for individual mortality outcomes would be stronger than for the composite outcome of 3P-MACE, as had been seen in EMPA-REG OUTCOME [48, 49]. Adding CV death as an outcome also presented an opportunity to demonstrate superiority in an individual CV outcome [48, 49]. However, the lack of superiority for all-cause mortality in the pooled data means that the subsequent analysis of CV death was exploratory only [17]. Similarly, subsequent analysis of CANVAS-R data alone, including superiority for albuminuria progression, composite of HHF and CV death, and CV death, also remain exploratory only [17].

#### Canagliflozin in subjects at increased CV risk

The significant 3P-MACE finding echoes that in EMPA-REG OUTCOME for empagliflozin; however, the lack of significance for both CV death and death by any cause was disappointing from a clinical point of view, as these parameters may be of most interest to clinicians, given the prevalent CV risk in patients with T2D [17].

The CANVAS Program study populations comprised 58.9% of patients with symptomatic CVD in CANVAS and 70.7% in CANVAS-R; the pooled figure was 65.6% [17]. These figures for CV involvement are more heterogeneous than the EMPA-REG OUTCOME population, where 99% of participants had established CVD [16]. Analysis of risk groups has shown that canagliflozin was superior to placebo for 3P-MACE in the secondary prevention group only, with no difference from placebo when looking only at primary prevention [46, 50]. However, when analysed for interactions between the primary, secondary and overall population, no statistically significant difference was seen (*p* = 0.18) [50].

The 35% of CANVAS Program patients who had CV risk factors but not symptomatic disease represent a challenge when interpreting the results, as the absence of symptoms does not necessarily represent an absence of disease, as evidenced by studies showing that people with T2D are known to have a high burden of asymptomatic CVD and that CAD may manifest in a silent fashion [51].

#### Renal outcomes in the CANVAS program

Analyses of renal outcomes in the CANVAS Program were exploratory only, owing to the failure to meet previous endpoints in the statistical hierarchy [17]. Nevertheless, exploratory findings with canagliflozin were promising, suggesting reductions not only in albuminuria, which we believe may not be the most robust metric for assessing effect on kidney function, but also in doubling of serum creatinine, which is a key marker of impaired renal function [34]. These suggested benefits are consistent with renal observations with empagliflozin treatment in EMPA-REG OUTCOME [33]. Renal burden in the CANVAS Program population was also reminiscent of EMPA-REG OUTCOME, with eGFR < 60 mL/min/1.73m^2^ in 20% of patients and 60–< 90 mL/min/1.73m^2^ in 55% of patients [52].

#### Key safety data from the CANVAS program

The safety results of note from the CANVAS Program showed an approximate two-fold increased risk of lower limb amputations with canagliflozin versus placebo (6.3 vs. 3.4 participants with amputation per 1000 patient-years: HR 1.97; 95% CI 1.41–2.75, *p* < 0.001) (Table 3), and confirmed a previous suggested increase in bone fractures (15.4 vs 11.9 participants with fracture per 1000 patient-years; HR, 1.26; 95% CI 1.04 to 1.52, *p* = 0.02) [17]. The only other event of significance in the CANVAS Program was an increase in genital infections in both men and women (*p* < 0.001), most likely due to the increased levels of glucose in the urine, and also seen with other SGLT2 inhibitors [17].

The incidence of lower limb amputation with canagliflozin was not uniform across all groups: patients with atherosclerosis and previous amputation had a higher risk of amputation compared with other patients [17]. Although amputations of the toe and middle of the foot were the most common, amputations involving the leg, below and above the knee, also occurred and some patients had more than one amputation, some involving both limbs [17].

In February 2017, the EMA issued a statement requiring a warning of the potential increased risk of toe amputation in the prescribing information for all SGLT2 inhibitors based on canagliflozin data, including from the CANVAS Program; however, the statement noted that an increased risk has not been seen to date in studies with empagliflozin or dapagliflozin [53]. Indeed, although amputation was not a pre-specified safety outcome of EMPA-REG OUTCOME, a post hoc analysis found no increased signal for amputation with empagliflozin compared with placebo [45]. Both the EMA and FDA have required the addition of a warning for increased risk of lower limb amputation to the canagliflozin label, but not to the empagliflozin or dapagliflozin labels [54].

The mechanisms by which canagliflozin may increase the risk of amputation are still unclear [54], especially as, we suggest, there does not appear to be a similar signal from empagliflozin or dapagliflozin. We believe that the increase in amputation rate is concerning, as the potential repercussions on quality of life are profound. It can be argued that many, if given the choice, would opt to accept an increased CV risk over a doubled risk of amputation.

### DECLARE-TIMI 58

The first results for the DECLARE-TIMI 58 CVOT on dapagliflozin have now been reported, enabling a more comprehensive analysis of CV and renal outcomes across the SGLT2 inhibitor class [18]. Although the data were disclosed subsequent to our initial discussions, and during the preparation of this manuscript, we believe that it is important to briefly address the findings here.

Notably, DECLARE-TIMI 58 is the first CVOT in the SGLT2 inhibitor class to include a majority of primary prevention patients; that is, 59% of patients had multiple CV risk factors (male aged ≥55 or female aged ≥60 with one or more of dyslipidaemia; hypertension; or current smoker), whereas only 41% of patients had established atherosclerotic CVD (age ≥ 40 with one or more of CAD; ischaemic cerebrovascular disease; or PAD) [18, 55].

In the initial study design, the primary safety endpoint was non-inferiority for 3P-MACE and the primary efficacy endpoint was superiority for 3P-MACE, as with EMPA-REG OUTCOME and the CANVAS Program. However, following EMPA-REG OUTCOME, a new co-primary composite efficacy endpoint of HHF and CV death was added, with permission from regulators, in light of the new insights that these outcomes may be highly clinically relevant with SGLT2 inhibitors [16, 18, 56].

As expected, DECLARE-TIMI 58 demonstrated that dapagliflozin was non-inferior to placebo as an add-on to standard of care in the study population, thus meeting the primary safety endpoint [18]. Similarly, DECLARE-TIMI 58 added further evidence that a reduction in HHF is consistent across the SGLT2 inhibitor class, as the co-primary efficacy endpoint of HHF or CV death was also met, and this was driven entirely by a decreased risk of HHF, while there was no difference between dapagliflozin and placebo in risk of CV death [18].

However, the study failed to meet the primary efficacy endpoint of superiority for 3P-MACE, with no significant difference between dapagliflozin and placebo [18]. Furthermore, even when looking only at patients with baseline established atherosclerotic CVD (a similar size cohort to EMPA-REG OUTCOME), there remained no significant benefit in either 3P-MACE or CV death with dapagliflozin [18, 46]. The risk of death by any cause was also not significantly reduced with dapagliflozin vs placebo [18].

### Considering EMPA-REG OUTCOME alongside the CANVAS Program and DECLARE-TIMI 58

Although direct comparisons can only be made between the effects of different agents in head-to-head studies, owing to differences in populations, trial designs, analytical approaches and drug effects, it can nevertheless be useful for clinicians to critically appraise data in the context of similar studies.

In a clinical setting, we believe that CV death could be considered to be the most relevant parameter for assessing the overall benefit from a glucose-lowering drug for patients with T2D, as reductions in CV death are the prime goal of treatment. In the EMPA-REG OUTCOME study, the RRR of CV death for empagliflozin vs placebo was 38% (HR 0.62; 95% CI 0.49–0.77, *p* < 0.001) [16]. By contrast, as described above, the RRR of CV death did not achieve statistical significance in either the CANVAS Program or in DECLARE-TIMI 58 [17, 18]. Thus, reduction in CV death was, among SGLT2 inhibitors, a unique finding with empagliflozin.

The results for HHF and renal outcomes were more similar between the three CVOTs. Whereas HHF is only presented as an exploratory outcome in the CANVAS Program (HR 0.67; 95% CI 0.52–0.87) and DECLARE-TIMI 58 (HR 0.73; 95% CI 0.61–0.88), the observed trend towards treatment benefit is similar to that seen with empagliflozin in EMPA-REG OUTCOME, where RRR for HHF was 35% (HR 0.65; 95% CI 0.50–0.85, *p* < 0.001) [16–18].

The design of composite renal outcomes differed between studies, and there is not a composite renal endpoint that has been reported for all three CVOTs. For the CANVAS Program, the key renal composite was a 40% reduction in eGFR, requirement for renal replacement therapy, or death from renal causes [17], while in DECLARE-TIMI 58 the composite comprised 40% reduction in eGFR to < 60 ml/min/1.73m^2^, end-stage renal disease, or death from renal causes [18]. Due to the failure to meet earlier endpoints, these renal outcomes were exploratory only in the two trials, but nevertheless both suggested a strong trend towards a protective effect with treatment, with 40% reduction in the CANVAS Program (HR 0.60; 95% CI 0.47–0.77) and 47% reduction in DECLARE-TIMI 58 (HR 0.53; 95% CI 0.43–0.66).

There was no equivalent pre-specified endpoint in EMPA-REG OUTCOME, although a post hoc analysis of doubling of serum creatinine accompanied by an eGFR of < 45 ml/min/1.73 m^2^, initiation of renal replacement therapy, or death due to renal disease is the closest approximate, and showed a similar benefit to the other CVOTs (46% reduction; HR 0.54; 95% CI 0.40–0.75) (Table 2) [33]. As noted above, results for the EMPA-REG OUTCOME prespecified composite of incident or worsening nephropathy, which also included progression to macroalbuminuria, were also similar (HR 0.61; 95% CI 0.53–0.70, *p* < 0.001) [16, 33]. An equivalent composite in the CANVAS Program yielded similar results in an exploratory analysis (HR 0.58; 95% CI 0.50–0.67) [34]. Albuminuria outcomes have not yet been reported for DECLARE-TIMI 58 [18].

In safety outcomes, much attention has been paid to the doubling of lower limb amputations in the CANVAS Program, in contrast to DECLARE-TIMI 58 and EMPA-REG OUTCOME, where no signal was seen (Table 3) [17, 18, 45]. Thus, canagliflozin is to date the only SGLT2 inhibitor that has produced a CVOT signal for lower limb amputation risk.

Bone fracture risk was also increased in the CANVAS Program but not EMPA-REG OUTCOME or DECLARE-TIMI 58, whereas the rare event of diabetic ketoacidosis (DKA) was significantly increased in DECLARE-TIMI 58 and had a trend towards an increase that did not reach significance in the CANVAS Program and EMPA-REG OUTCOME (Table 3) [16–18]. The prescribing information for all SGLT2 inhibitors cautions prescribers to be alert for signs of DKA, which although rare is potentially dangerous and may present atypically (that is, in patients with only moderately increased blood glucose values) [36].

There was a mixed picture for urogenital infections in SGLT2 inhibitor CVOTs. While there was a consistent signal for increased risk of genital infections with all three agents, which was also seen in previous trials [16–18], none of the CVOTs showed an increase risk of urinary tract infection (UTI), although a signal was seen with empagliflozin when looking at female patients alone [16]. Other studies have shown inconsistent results for UTI risk with SGLT2 inhibitors, with four recent meta-analyses finding no evidence for increased risk of UTI with any agent in the class, with the possible exception of high dose (10 mg) dapagliflozin [57–60]. Nevertheless, prescribers should be advised that current product labels contain a warning about possible UTI events [36].

To summarise the SGLT2 CVOT data, despite the recent CANVAS Program and DECLARE-TIMI 58 results, empagliflozin remains the only member of the SGLT2 inhibitor class thus far having proven a significant reduction in CV death (38% RRR) in a dedicated and robust CVOT that was designed, and powered, to test for superiority in CV outcomes versus placebo [16–18]. Furthermore, the reduced risk of CV death with empagliflozin was consistent across pre-specified analyses [16]. By contrast, there was no significant reduction in CV death versus placebo with either canagliflozin, in the CANVAS Program, or dapagliflozin, in DECLARE-TIMI 58, and this was true whether analysing each study population as a whole or only the patients with baseline symptomatic atherosclerotic CVD [17, 18, 46, 50]. However, HHF and renal endpoints suggested that all SGLT2 inhibitors provided a benefit for these outcomes, although these analyses were exploratory only in the CANVAS Program and DECLARE-TIMI 58 due to the hierarchical statistical testing plan design [16–18, 33, 34]. Safety outcomes also showed differences, with increased risk of lower limb amputation and bone fracture in the CANVAS Program, but not EMPA-REG OUTCOME or DECLARE-TIMI 58, although an increased risk of genital infections was consistent across the class, and prescribers should be advised of the possibility of rare DKA events with all three agents [16–18, 36, 45].

One possible explanation for different outcomes between SGLT2 inhibitor CVOTs may be differences in study design, some of which are outlined above. However, another explanation may be the differences in molecular structure that result in different relative selectivity for SGLT2 over SGLT1 [32]. SGLT1 inhibition is known to cause gastrointestinal problems and investigations into the earliest SGLT2 inhibitors were abandoned owing to their lack of selectivity between SGLT1 and SGLT2 [61]. It may be that there are other repercussions of the different molecular structures that have not yet been identified.

### Real-world evidence

In recent years, RWE has begun to provide insights into clinical and health economic outcomes with SGLT2 inhibitors in routine clinical practice, and ongoing RWE studies are set to shed still further light on this in the years to come. RWE is captured in natural, uncontrolled settings outside of traditional RCTs, and has been said to “represent a measure in understanding healthcare data collected under real-life practice circumstances” [62]. RWE studies can provide data on effectiveness and safety during routine care, complement and support data from RCTs, and support market access and reimbursement decisions [62]. However, RWE alone is insufficient to demonstrate efficacy, and thus cannot be used on its own to meet regulatory requirements for passing a new indication or extending an existing indication [63]. RWE is commonly used for patient profiling and prevalence, defining treatment pathways and patterns of care, evaluating compliance and persistence, determining treatment costs and costs in disease stages and disease states, and informing investigations on health outcomes and disease sequelae [62].

#### CVD-REAL

CVD-REAL was a RWE study that compared the rate of HHF in individuals with T2D who had been newly initiated on SGLT2 inhibitors (canagliflozin, dapagliflozin or empagliflozin) versus other glucose-lowering drugs (oGLDs), with secondary aims of comparing the risk of all-cause death, and HHF or all-cause death, between the two treatment groups [29]. The cohort included both patients with and without established CVD at baseline [29]. For patients without CVD at baseline, HF may be a key outcome, as it was shown to be among the most common first presentations of CVD in a prospective cohort study of health records over 5.5 years (14.1% of patients with T2D) [64].

Data for CVD-REAL were gathered from registries and national initiatives from six different countries (US, UK and Nordic countries). From an initial post-screening population of 160,033 people who had been prescribed an SGLT2 inhibitor and 1,226,221 who had been given an oGLD, subjects were propensity matched 1:1 to give a test population of 154,528 for each of SGLT2 inhibitors or oGLDs [29]. This propensity matching step ensured that the baseline characteristics were similar for each group, although they did differ somewhat from CVOTs: for example, patients were relatively young (57 years vs 63–64 years in CVOTs); fewer (13%) had prior CVD; statin use was relatively high at 67%, but slightly less than in CVOTs; and concomitant GLP-1 receptor agonists were much more widely used (18–20% in CVD-REAL vs 3–5% in SGLT2 inhibitor CVOTs) [16–18, 29]. Overall, 53% of the study population received canagliflozin, 37% received dapagliflozin and 10% received empagliflozin. These percentages varied by region: in the US, 75.9% received canagliflozin, whereas in Europe 91.9% received dapagliflozin. All three primary analyses favoured SGLT2 inhibitors over oGLD: HHF (HR 0.61; 95% CI 0.51–0.73, *p* < 0.001); death by any cause (HR 0.49; 95% CI 0.41–0.57, *p* < 0.001), and a composite of HHF or death by any cause (HR 0.54; 95% CI 0.48–0.60, *p* < 0.001) [29]. Results for individual SGLT2 inhibitors were not reported.

The related study CVD-REAL 2 used a similar study design to look at CV and mortality outcomes in real-world data from an additional six countries (four from Asia Pacific, plus Canada and Israel), with 235,000 treatment initiations in each study arm [65]. In this second study, 75% of patients received dapagliflozin, with the remainder split between empagliflozin (9%), canagliflozin (4%), and three additional SGLT2 inhibitors that are not currently licensed in the European Union (12%) [65]. Results in CVD-REAL 2 were similar to CVD-REAL, with a lower incidence of the composite of HHF or death by any cause (HR 0.60; 95% CI 0.47–0.76, *p* < 0.001), as well as a lower incidence of the individual components HHF (HR 0.64; 95% CI 0.50–0.82, *p* = 0.001) and death by any cause (HR 0.51; 95% CI 0.37–0.70, *p* < 0.001) [65].

#### EASEL

The EASEL study was a retrospective cohort study of SGLT2 inhibitors in patients with T2D and CVD that used data from the US Department of Defense (DoD) Military Health System (MHS) [66]. The study found that patients newly initiated on SGLT2 inhibitors had 43% fewer HHF or death by any cause events than propensity matched patients initiated on non-SGLT2 inhibitor therapy (1.73 versus 3.01 composite events per 100 person-years; HR, 0.57; 95% CI, 0.50–0.65) [66]. Similarly, the number of MACE events was 33% lower in the SGLT2 inhibitor cohort than in the propensity matched non-SGLT2 inhibitor cohort (2.31 versus 3.45 events per 100 person-years; HR, 0.67; 95% CI, 0.60–0.75). However, pooled safety data showed that SGLT2 inhibitors were associated with an approximately 2-fold higher risk of below knee lower extremity amputation, similar to the risk observed with canagliflozin in the CANVAS Program [17, 66]. The amputation rates for the individual agents varied slightly, with canagliflozin showing a slightly higher incidence rate than empagliflozin or dapagliflozin, after propensity matching [66].

#### EMPRISE

During the preparation of this manuscript, early results from an additional RWE study were announced. The study, EMPRISE, uses 3 large US databases to assess outcomes with empagliflozin in routine clinical practice, with DPP-4 inhibitors as an active comparator [30]. EMPRISE will assess a range of effectiveness, safety, healthcare resource utilisation and cost outcomes, in a patient population with a much broader CV risk than in EMPA-REG OUTCOME [30]. By the time of study completion, it is envisaged that 232,000 patients will have been included over the course of 5 years, with 116,000 patients in each propensity matched arm [30]. Recently, initial effectiveness results after 5 months follow-up were disclosed for selected outcomes in the first 35,000 patients [30]. These results suggested that patients receiving empagliflozin had substantially fewer HHF events than patients receiving DPP-4 inhibitors (HR 0.49; 95% CI 0.27–0.89) [30]. Thus, preliminary results from EMPRISE are reminiscent of the rapid benefit seen with empagliflozin in reducing HHF events in the controlled conditions of EMPA-REG OUTCOME [16, 43].

#### RWE data in context

We believe that the value of RWE must be cautiously taken in the context of evidence from CVOTs, as data generated from observational studies are not comparable to gold standard RCTs, and residual confounding such as selection bias cannot be excluded. For example, no information is available on the safety profile of SGLT2 inhibitors within CVD-REAL, nor on CV risk, making it difficult to draw conclusions regarding risk:benefit profiles. Of particular note, the reductions in all-cause mortality reported with pooled SGLT2 inhibitors in CVD-REAL were not replicated for canagliflozin in the CANVAS Program, nor for dapagliflozin in DECLARE-TIMI 58 [17, 18, 29]. Indeed, there is a possible inherent bias in the CVD-REAL design, due to the hierarchical nature of therapy for T2D, in which patients are generally prescribed SGLT2 inhibitors only after therapy with oGLD. This means that for such patients the oGLD treatment period becomes defined as “immortal”, as patients who are subsequently prescribed SGLT2 inhibitors, by definition, have survived [29, 67]. However, we note that the authors of the study have argued against the potential of such a bias to affect the results [68], and as such the subject remains a matter of debate [69]. Concerns over the possibility of immortal time bias have been addressed in the study design for EMPRISE, where patients are matched for number of diabetes therapies and the study is designed to compare treatments with the same position in the treatment pathway [30].

It is our opinion that RWE studies can be of use where the data support and agree with results from RCTs. For example, the evidence from RWE supports the findings from CVOTs showing a reduction in HHF with SGLT2 inhibitors, which suggests that the reduction in HHF demonstrated in CVOTs may be observed in T2D patients across a broad continuum of CVD in routine clinical practice.

### SGLT2 inhibitors and class effect — what is the evidence from CVOTs and RWE?

Taking into consideration the various results from SGLT2 inhibitor CVOTs, we consider that it is still too early to safely assume a class effect. As such, we believe that each agent should be evaluated according to its individual data and merit.

In particular, we feel that the different results in CV death, death by any cause and safety cast doubt on the potential extent of a class effect, despite similar reductions in HHF and renal outcomes (and exploratory findings) between the three CVOTs. We note that variability across a class in CV death outcomes has a clear precedent in CVOTs: among GLP-1 receptor agonist CVOTs, a reduction in CV death was seen with liraglutide, but not with lixisenatide, exenatide, semaglutide or the unlicensed agent albiglutide [23–26, 70].

International guidelines that have been updated in the light of CVOTs have recognised this distinction, recommending an SGLT2 inhibitor or GLP-1 receptor agonist with proven CV benefit for patients with T2D in an atherosclerotic CVD setting [37–39]; or an SGLT2 inhibitor with proven HF or CKD benefit in patients where HF or CKD predominates [37, 38].

## Conclusions

CVOTs investigating SGLT2 inhibitors have suggested benefits beyond glucose lowering that have been confirmed in RWE studies [16–18, 29, 30, 33, 34, 65, 66], which has led guidelines to support a favourable positioning for these agents early in the treatment pathway for patients with T2D in the setting of CV risk, HF and renal disease [37–41]. However, empagliflozin is the only drug within this class to have demonstrated proven efficacy and safety across the most relevant endpoints, namely CV death and death by any cause, as well as other CV and renal outcomes [16–18].

## Data Availability

Data sharing is not applicable to this article as no datasets were generated or analysed during the current study. All data discussed were taken from the published sources cited.

## Abbreviations

3/4P-MACE: 3/4-point MACE
ADA: American Diabetes Association
CI: Confidence interval
CV: Cardiovascular
CVD: CV disease
CVOT: CV outcomes trial
eGFR: Estimated glomerular filtration rate
EMA: European Medicines Agency
FDA: US Food and Drug Administration
GLP-1: Glucagon-like peptide-1
HF: Heart failure
HHF: Hospitalisation for heart failure
HR: Hazard ratio
MACE: Major adverse CV event
oGLD: Other glucose-lowering drug
PAD: Peripheral artery disease
RCT: Randomised controlled trial
RRR: Relative risk reduction
RWE: Real-world evidence
SGLT1/2: Sodium–glucose transporter 1/2
T1/2D: Type 1/2 diabetes
UACR: Urine albumin-to-creatinine ratio
